# Glycerol monolaurate attenuates necrotic enteritis in broilers by improving gut–liver health and remodeling the gut microbiota

**DOI:** 10.1186/s40104-026-01388-w

**Published:** 2026-04-18

**Authors:** Linglian Kong, Xinran Zhang, Xue Pan, Xuewei Dong, Zhigang Song

**Affiliations:** 1 Development Planning Office, Jining Polytechnic, Jining, 272037 China; 2 Key Laboratory of Efficient Utilization of Non-Grain Feed Resources (Co-Construction By Ministry and Province), Ministry of Agriculture and Rural Affairs, Shandong Provincial Key Laboratory of Animal Nutrition and Efficient Feeding, Department of Animal Science, Shandong Agricultural University, Taian, 271018 China; 3 College of Geodesy and Geomatics, Shandong University of Science and Technology, Qingdao, 266590 China; 4 College of Animal Science, Wenzhou Science and Technology Vocational College, Wenzhou, 325006 China

**Keywords:** Glycerol monolaurate, Gut microbiota, Intestinal inflammation, Necrotic enteritis, Oxidative stress

## Abstract

**Background:**

Necrotic enteritis (NE) is a widespread avian intestinal disease that causes substantial economic losses in poultry production. With increasing restrictions on antibiotic use, sustainable alternatives are urgently needed. Glycerol monolaurate (GML) has anti-inflammatory and antimicrobial properties, but its role in preventing NE remains unclear. This study aimed to investigate the protective effects of GML on gut–liver health and the cecal microbiota in NE-challenged broilers.

**Results:**

A total of 288 broilers were fed diets supplemented with 1,200 mg/kg GML for 21 d under an NE challenge model. GML improved the average daily gain and jejunal villus height while reducing the intestinal lesion score in NE broilers (*P* < 0.05). GML upregulated the expression of the tight junction genes *OCLN* and *MUC2* and downregulated the expression of the proinflammatory cytokines interleukin (*IL*)-*1β*, *IL*-*8*, and tumor necrosis factor (*TNF*)-*α*, as well as *RELA* and *IRF5* (*P* < 0.05). Moreover, GML reduced endotoxin levels and alleviated oxidative stress in the jejunum of NE broilers. NE infection increases intestinal permeability, resulting in elevated serum proinflammatory cytokine (IL-1β, TNF-α) and intestinal-derived endotoxin levels, which subsequently contribute to liver injury. GML significantly alleviated this liver damage, as evidenced by reduced hepatic steatosis and lower serum alanine aminotransferase levels (*P* < 0.05). Furthermore, GML relieved the NE-induced increase in serum endotoxin and proinflammatory cytokine levels and improved the hepatic antioxidant status by decreasing the malondialdehyde content and increasing superoxide dismutase activity (*P* < 0.05). Additionally, GML significantly enhanced the alpha diversity indices and increased the relative abundances of *Parabacteroides*, *Lactobacillus*, *Blautia*, and *DTU089* in the ceca of NE broilers, with these microbial changes showing significant correlations with improvements in inflammatory and oxidative status. GML altered microbial functions in NE broilers, affecting pathways related to the biosynthesis of unsaturated fatty acids, antibiotic biosynthesis, and phosphonate and phosphinate metabolism. These functional shifts support the potential mechanisms through which the altered microbiota may contribute to the observed improvements.

**Conclusions:**

In summary, GML supplementation alleviated NE in broilers by enhancing intestinal barrier function, reducing inflammation and oxidative stress, and modulating the gut microbiota composition, thereby attenuating gut-derived liver injury and improving growth performance.

## Background

Necrotic enteritis (NE) is a globally prevalent intestinal disorder in poultry caused by *Clostridium perfringens*, resulting in substantial economic losses in the poultry sector [1]. This disease negatively impacts the growth performance of broilers by inducing chronic damage to the intestinal lining and impairing nutrient uptake [2]. Growing evidence indicates that the development of NE is strongly linked to impairment of the intestinal barrier [3]. The intestinal barrier is primarily maintained by tight junction proteins, including claudin-1, occludin, and zonula occludens-1 (ZO-1), which form a selective permeability barrier regulating the paracellular transport of ions, nutrients, and macromolecules. The compromise of the intestinal epithelial barrier allows the passage of detrimental pathogens and endotoxins (ET) from the gut lumen into the surrounding tissues, which triggers mucosal injury and inflammatory responses within the intestine [4]. Moreover, gut-derived endotoxins can enter the portal circulation and induce liver injury via the gut-liver axis, a key physiological pathway in which the liver serves as the first line of defense for blood draining from the gut. This hepatic involvement exacerbates the progression of NE by promoting cholestasis and increasing the levels of inflammatory factors in the circulation [5]. Antibiotics have historically been used as effective means of controlling NE in broilers. However, restrictions on antibiotic growth promoters in many countries have created obstacles for intensive broiler production, which is correlated with increased NE incidence rates [6]. More seriously, over 94.5% of *Clostridium perfringens* isolates exhibit multidrug resistance, which not only compromises the effectiveness of conventional antibiotic therapies but also poses a major threat to public health [7]. Therefore, the poultry industry urgently needs effective alternatives to antibiotics for the prevention of NE [8].

Glycerol monolaurate (GML) is a 12-carbon molecule of natural origin that is present in breast milk, coconut oil and palm oil. Previous research has identified GML as an innovative, eco-friendly, and highly potent antimicrobial compound that has been demonstrated to be safe for broilers at dosages up to 5 g/kg [9]. The amphiphilic structure of GML, comprising a hydrophilic glycerol head and a lipophilic lauric acid tail, enables the lipophilic tail of the molecule to be inserted into the lipid envelope of Gram-positive bacterial cell membranes. This insertion disrupts membrane integrity, compromises ion exchange, and ultimately leads to cell death, explaining why GML is especially effective against Gram-positive pathogens such as *Clostridium perfringens*. In addition to this direct antimicrobial action, GML functions as a signaling molecule that exerts anti-inflammatory, antioxidant, and immunomodulatory effects, playing a crucial role in alleviating intestinal inflammation. The dietary inclusion of GML enhances intestinal barrier integrity and reduces hepatic steatosis in laying hens under heat stress, alleviating associated oxidative damage and lipid metabolism disturbances [10]. Its efficacy in avian species has been further demonstrated by in ovo studies, in which GML administration improved the intestinal morphology and antioxidant status of chicken embryos by suppressing inflammatory responses and inhibiting nuclear factor kappa-B (NF-κB) signaling [11]. Moreover, GML has synergistic protective effects against NE in broilers when combined with cinnamaldehyde [12]. Complementary support arises from studies in other monogastrics, such as weaned piglets, where GML improves the intestinal structure and immune function [13].

Given that gut microbiota dysbiosis is strongly implicated in the pathogenesis of NE in broilers [14, 15], modulating the intestinal ecosystem through feed additives has emerged as a promising preventive strategy [16]. Mechanistic insights from murine models indicate that GML alleviates colitis and corrects microbiota dysbiosis, with its protective effects being transmissible via fecal microbiota transplantation [17, 18]. On the basis of these findings and promising avian-specific results [11, 12], we hypothesized that dietary GML would ameliorate NE by enhancing intestinal barrier function, attenuating gut-derived liver injury, and restoring microbial homeostasis. However, while the antimicrobial properties of GML have been documented, its capacity to mediate the bidirectional communication between the gut and the liver during localized intestinal infection remains unexplored. This study therefore evaluated the effects of dietary GML on growth performance, intestinal morphology, inflammatory and oxidative status, liver health, and the cecal microbiota in broilers challenged with *Clostridium perfringens*. These findings provide a theoretical foundation for leveraging GML in the prevention of NE in broilers.

## Materials and methods

### Birds, diets, and experimental design

A total of 288 one-day-old male Arbor Acres broilers were randomly allocated into four experimental groups: a control group (CON), a GML diet group (GML), an NE infection group (NE), and a group that received a GML diet and NE infection (GN). Each group consisted of 6 replicate pens, with 12 birds housed per pen. The GML used, which had a purity of 90%, was provided by Zhengtong Food Technology Co., Ltd. (Zhengzhou, Henan, China) and was incorporated into the basal diet at a concentration of 1,200 mg/kg. This dosage was selected on the basis of a previous study [19]. To ensure homogeneous distribution, GML was first premixed with a small portion of the basal diet, followed by gradual blending with the remaining feed using a mechanical mixer. The diets were prepared weekly and stored in a cool, dry place. The basal corn-soybean meal diets (Table 1) were formulated in accordance with the nutritional guidelines for Arbor Acres broilers [21]. Metabolizable energy values for the basal diets were calculated via feed composition and nutrient data from the China Feed Database [20]. The crude protein content was determined via the Kjeldahl method following GB/T 6432–2018 [22]. Calcium levels were assessed via potassium permanganate titration according to GB/T 6436–2018 [23], whereas total phosphorus was measured via spectrophotometric analysis in line with GB/T 6437–2018 [24]. Methionine and lysine concentrations were quantified using ninhydrin derivatization on the basis of GB/T 18246–2019 [25]. The feed was provided in powder form throughout the entire trial period. This form was chosen specifically to ensure the precise and homogeneous distribution of the supplemented GML throughout the basal diet, thereby guaranteeing consistent additive intake across all birds within a treatment group. The birds were housed in an environmentally controlled chamber with unrestricted access to feed and water. The chamber was maintained at 60% ± 10% relative humidity, with a lighting schedule consisting of 23 h of light followed by 1 h of darkness. The initial ambient temperature was set at 33 °C and was subsequently decreased by 3 °C per week until it reached 25 °C.

**Table 1:** Composition and nutrient level of the basal diet (as-fed basis), %

Ingredients	Content	Nutrient levels^2^	Content
Corn	63.19	Metabolic energy, MJ/kg	12.55
Soybean meal (43%)	28.77	Dry matter	88.50
Soybean oil	3.29	Crude protein	21.0
Corn protein flour (60%)	1.54	Calcium	0.90
Limestone	1.13	Total phosphorus	0.65
Dicalcium phosphate	0.57	Available phosphorus	0.45
Salt	0.26	Lysine	1.25
DL-Methionine	0.23	Methionine	0.55
L-Lysine HCl (99%)	0.26	Methionine + Cysteine	0.95
L-Threonine (98.5%)	0.12	Threonine	0.85
L-Tryptophan	0.02	Tryptophan	0.24
Choline chloride (50%)	0.15		
Phytase (20,000 U)	0.02		
Sodium bicarbonate	0.14		
Premix^1^	0.31		
Total	100.00		

^1^Provided per kilogram of diet: vitamin A, 12,000 IU; vitamin D_3_, 3,500 IU; vitamin E, 30 mg; vitamin K_3_, 3.0 mg; vitamin B_1_, 2.0 mg; vitamin B_2_, 8.0 mg; vitamin B_6_, vitamin B_12_, 0.02 mg; 5.0 mg; nicotinic acid, 50 mg; pantothenic acid, 15 mg; biotin, 0.15 mg; folic acid, 2.0 mg; I, 1.0 mg; Fe, 80 mg; Mn, 100 mg; Se, 0.3 mg; Zn, 100 mg; Cu, 8 mg

^2^Metabolizable energy was calculated on the basis of Tables of Feed Composition and Nutritional Values in China (China Feed Database, [20]), while all other nutrient levels were analyzed

A two-factor completely randomized design was employed to explore the impact of GML on NE infection in broilers. Dietary levels of GML were regarded as one factor, and NE infection was viewed as another contributing element. The previously reported NE model was employed in the present study with certain modifications [26, 27]. Specifically, birds in the NE and GN groups were orally administered 1 mL of coccidia vaccine (Zhengdian Biotechnology Co., Ltd., Foshan, China) at 14 days of age, whereas birds in the CON and GML groups received an equivalent volume of sterile phosphate-buffered saline. The 1 mL vaccine contained 1 × 10^4^ sporulated oocysts of *E. tenella* strain PTMZ, *E. necatrix* strain PNHZ, *E. maxima* strain PMHY, and *E. acervulina* strain PAHY. From 18 to 20 days of age, *Clostridium perfringens* (CVCC52, National Center for Veterinary Culture Collection, China Institute of Veterinary Drug Control, Beijing, China) was administered at a concentration of 5 × 10^8^ CFU/mL by oral gavage daily (1 mL) to birds in the NE and GN groups, whereas those in the CON and GML groups were given the same volume of sterile *Clostridium perfringens* culture medium. The bacterial suspension was prepared daily as follows: Briefly, the strain stored at −80 °C was first activated on tryptone-sulfite-cycloserine agar plates (Hope Bio-Technology Co., Ltd., Qingdao, China) under anaerobic conditions at 37 °C for 24 h. A single colony was then inoculated into 10 mL of sterile reinforced clostridial medium (Hope Bio-Technology Co., Ltd., Qingdao, China) and incubated anaerobically at 37 °C for 18 h as the primary culture. Subsequently, 1 mL of this primary culture was transferred into 300 mL of fresh sterile reinforced clostridial medium and incubated anaerobically at 37 °C until it reached the mid-logarithmic growth phase, yielding a final inoculum with a concentration of 5 × 10^8^ CFU/mL. The optical density at OD_600_ was monitored to confirm the growth phase.

### Growth performance

On d 21, the body weights and feed weights of the birds were measured after they had fasted for 12 h. Any feed that had been spilled was meticulously collected and weighed to precisely adjust the final feed intake figures. The average daily feed intake (ADFI), average daily gain (ADG), and feed conversion ratio (FCR) were calculated for the periods from 1 to 21 d.

### Sample collection and jejunal lesion scoring

The birds were euthanized and sampled at 21 days of age. This timepoint, approximately 24 h after the final *Clostridium perfringens* inoculation, was selected to assess the acute phase response to the NE challenge and its modulation by dietary GML, in accordance with established protocols for this model [28]. One bird per replicate was randomly chosen, and blood was drawn from its wing veins. The collected blood samples were centrifuged at 3,000 × *g* for 10 min at 4 °C to separate the serum, which was then preserved at −20 °C for later analysis. The birds were humanely euthanized by cervical dislocation, after which the liver, jejunum, and ceca were promptly and carefully excised. To ensure a representative assessment of intestinal morphology, multiple adjacent segments, each approximately 1 cm in length, were excised from the visibly affected jejunal region, spanning from the bile duct entry point to Meckel's diverticulum, and subsequently examined. The segment that appeared most representative of the overall intestinal condition was selected, rinsed with sterile saline, and fixed in 4% paraformaldehyde. Sections of the liver and jejunum, as well as the entire ceca, were removed and rapidly frozen in liquid nitrogen before being stored at −80 °C for subsequent analysis. Lesion scoring of the jejunum was performed via visual observation using a previously established 4-point lesion scoring system [29], where 0 indicated no lesions and 4 represented the most severe macroscopic lesions.

### Liver morphology analysis

For Oil Red O staining, liver samples were embedded in optimal cutting temperature compound and sliced into 5 μm sections using a Leica CM1950 microtome (Leica, Wetzlar, Germany). The sections were fixed in 4% paraformaldehyde for 10 min, rinsed with distilled water and then washed with 60% isopropanol. Then, the sections were stained with Oil Red O for 30 min, rinsed again with distilled water, and briefly counterstained with hematoxylin. After a final wash with distilled water, the sections were mounted with glycerin and observed under a microscope (Leica DM IL LED, Wetzlar, Germany). Images were acquired at 200 × magnification.

### Oxidative status assay

Samples of jejunum and liver tissues were homogenized in sterile phosphate-buffered saline to prepare a 10% (w/v) suspension. This suspension was then centrifuged at 1,000 × *g* for 10 min at 4 °C. The obtained supernatant was collected for the determination of malondialdehyde (MDA) levels and the activities of superoxide dismutase (SOD) and catalase (CAT). Commercial assay kits were supplied by Nanjing Jiancheng Biotechnology Institute (Nanjing, China). The intra-assay coefficients of variation were maintained below 5%, and the interassay coefficients of variation were under 8%. The results for each sample were normalized to the total protein concentration in the supernatant, as measured by a bicinchoninic acid protein assay kit (Beyotime Biotechnology, Nanjing, China).

### Assessment of intestinal barrier integrity

Jejunal tissues were fixed in a 4% paraformaldehyde solution for 24 h, after which they were dehydrated and embedded in paraffin. The tissue sections were subsequently sliced to a thickness of 4 μm and stained with hematoxylin‒eosin (H&E). The slices were then examined with an optical microscope (Nikon Eclipse 80i, Tokyo, Japan), with images captured at 400 × magnification. Villus height (VH) and crypt depth (CD) were determined, and the ratio of villus height to crypt depth was calculated.

Total RNA was extracted from jejunal tissue samples via TRIzol reagent (Invitrogen, Waltham, MA, USA). The mRNA expression levels of genes encoding critical tight junction proteins, including ZO-1 (*TJP1*), occludin (*OCLN*), and claudin-1 (*CLDN1*), were determined by quantitative real-time polymerase chain reaction (qRT-PCR) via gene-specific primers and normalized to *β-actin*.

### Endotoxin and cytokine detection assay

The concentrations of ET, interleukin (IL)-1β, IL-6, and tumor necrosis factor (TNF)-α in the homogenized jejunal supernatant and serum samples were quantified using enzyme-linked immunosorbent assay (ELISA) kits according to the manufacturer’s protocols (Shanghai Enzyme-linked Biotechnology Co., Ltd., Shanghai, China). The coefficients of variation for both the intra-assay and inter-assay assessments were kept under 10%.

### Analysis of AST and ALT levels

Serum aspartate aminotransferase (AST) and alanine aminotransferase (ALT) levels were measured by ELISA kits (Shanghai Enzyme-linked Biotechnology Co., Ltd., Shanghai, China) following the protocols provided by the manufacturer. The coefficients of variation for both the intra-assay and inter-assay assessments were maintained below 10% throughout the ELISA testing.

### RNA isolation and qRT-PCR assay

Total RNA was extracted from the jejunum using TRIzol reagent (Invitrogen, Waltham, MA, USA). Subsequently, 1 μg of the isolated RNA was reverse transcribed via a reverse transcription kit (AG11728, Accurate Biotechnology (Hunan) Co., Ltd., Changsha, China). qRT-PCR analyses were conducted in triplicate using targeted primers and SYBR Green Premix Ex Taq (AG11718, Accurate Biotechnology) on an ABI-7500 System (Thermo Fisher Scientific, Waltham, MA, USA). All primers were validated prior to qRT-PCR, and their sequences are detailed in Table 2. The amplification efficiency for each primer pair was assessed by generating standard curve dilutions, and the specificity was confirmed through melt curve analysis. Target gene expression levels were quantified relative to those of glyceraldehyde-3-phosphate dehydrogenase (*GAPDH*) using the 2^−ΔΔCt^ method.

**Table 2:** Primer sequences used for real-time quantitative PCR

Gene	Accession number	Primer sequence (5′→3′)	Product size, bp
*TJP1*	XM_015278981.2	CTTCAGGTGTTTCTCTTCCTCCTCTC	131
		CTGTGGTTTCATGGCTGGATC	
*OCLN*	NM_205128.1	TCATCGCCTCCATCGTCTAC	142
		TCTTACTGCGCGTCTTCTGG	
*CLDN1*	NM_001013611.2	CTGATTGCTTCCAACCAG	140
		CAGGTCAAACAGAGGTACAAG	
*MUC2*	NM_001318434.1	AGGAATGGGCTGCAAGAGAC	77
		GTGACATCAGGGCACACAGA	
*IL-1β*	NM_204524.1	GGTCAACATCGCCACCTACA	131
		CATACGAGATGCAAACCAGCAA	
*IL-6*	NM_204628.1	AAATCCCTCCTCGCCAATCT	106
		CCCTCACGGTCTTCTCCATAAA	
*IL-8*	NM_205498.1	GGAGTGCTCTAGTATGTTGTGT	119
		ACCCACAGTCTTACAGATTCTG	
*TNF-α*	NM_204267.1	TGTGTATGTGCAGCAACCCGTAGT	229
		GGCATTGCAATTTGGACAGAAGT	
*RELA*	NM_001396038.1	CAGCCCATCTATGACAACCG	152
		TCAGCCCAGAAACGAACCTC	
*TLR4*	NM_001030693.1	AGGCACCTGAGCTTTTCCTC	96
		TACCAACGTGAGGTTGAGCC	
*IRF5*	NM_001031587.2	GAGGGAGAGGAGGATCCAAG	219
		GACAGCTCCCCTGAGAACAG	
*TGF-β1*	NM_001318456.1	GATGGACCCGATGAGTATTG	118
		CGTTGAACACGAAGAAGATG	
*GAPDH*	AF047874	GAAGGCTGGGGCTCATCTG	150
		CAGTTGGTGGTGCACGATG	

*TJP1* Zonula occludens 1, *OCLN* Occludin, *CLDN1* Claudin 1, *MUC2* Mucin 2, *IL-1β* Interleukin 1 beta, *IL-6* Interleukin 6, *IL-8* Interleukin 8, *TNF-α* Tumor necrosis factor alpha, *RELA* Nuclear factor kappa-B p65, *TLR4* Toll-like receptor 4, *IRF5* Interferon regulatory factor 5, *TGF-β1* Transforming growth factor beta 1, *GAPDH* Glyceraldehyde-3-phosphate dehydrogenase

### 16S rRNA sequencing and analysis

Genomic DNA was isolated from the cecal contents using the E.Z.N.A.^®^ Soil DNA Kit (Omega Bio-Tek, Norcross, CA, USA) following the manufacturer's instructions. The DNA concentration and purity were evaluated via a Nanodrop 2000 spectrophotometer (Thermo Fisher Scientific, MA, USA), and the DNA was subsequently diluted to a final concentration of 1 ng/μL. The V3–V4 hypervariable regions of the 16S rRNA gene were amplified via specific composite primers (338F and 806R). The PCR cycling conditions included initial denaturation at 95 °C for 3 min, followed by 27 cycles of denaturation at 95 °C for 30 s and annealing/extension at 72 °C for 45 s, with a final elongation step at 72 °C for 10 min. The amplified products were verified by 2% agarose gel electrophoresis and then purified via a Universal DNA Purification Kit (Tiangen Biotech, Beijing, China). The NEBNext^®^ Ultra DNA Library Prep Kit for Illumina was used for library construction (New England Biolabs, MA, USA), and sequencing was conducted on the Illumina NovaSeq 6000 platform in PE250 mode (Illumina, CA, USA). All steps mentioned above were conducted by Gene Denovo Biotechnology Co., Ltd. (Guangzhou, China).

Bioinformatic analysis and visualization were performed via the Gene–Denovo Cloud Platform (https://www.omicsmart.com). The raw data were processed using FASTP (version 0.18.0) to remove reads containing ≥ 10% ambiguous (N) bases, reads with ≥ 50% of bases having a Phred quality score ≤ 20, or reads containing adapter sequences. Clean reads were merged into tags via FLASH (version 1.2.11) with a minimum overlap of 10 bp and a maximum mismatch rate of 2%. Tags were truncated at the first low-quality base (Q ≤ 3) within a 3-bp sliding window, and tags with high-quality regions comprising less than 75% of the original length were discarded. USEARCH (version 11.0.667) was employed to cluster the resulting clean tags into operational taxonomic units (OTUs) at 97% similarity via the UPARSE algorithm, followed by chimera removal with UCHIME. The resulting effective tags were used for OTU abundance analysis, and representative sequences were taxonomically annotated against the SILVA database (version 138.2) using the RDP classifier. The samples were rarefied to a sequencing depth of 10,000 reads per sample, a level confirmed to be sufficient on the basis of rarefaction curve analysis. Microbiota α diversity was computed using the Chao1, Sobs, ACE, Good’s Coverage, PD-whole tree, Pielou, Shannon and Simpson indices with the vegan package (version 2.6–4) in R (version 4.0.4) using RStudio (version 1.4.1106; RStudio, PBC, Boston, MA, USA). Beta diversity patterns were visualized through principal coordinate analysis (PCoA), with group-level differences assessed by Adonis and analysis of similarities (ANOSIM) tests. To identify bacterial taxa exhibiting significant variation, multiple methods were applied, including random forest classification, indicator species analysis, linear discriminant analysis effect size (LEfSe), Wilcoxon rank-sum testing, and receiver operating characteristic (ROC) curve evaluation. The functional profile of the gut microbiome was predicted via PICRUSt2, referencing level 2 and 3 pathways from the KEGG database. Spearman’s rank correlation analysis was used to investigate the relationships between various bacterial taxa and biomarkers indicative of inflammation and oxidative stress.

### Statistical analysis

For growth performance, the pen was the experimental unit; for all other parameters, the individual bird selected per pen was considered the experimental unit. All the statistical analyses were performed using SPSS software (IBM SPSS Statistics 26.0; Armonk, NY, USA). Prior to hypothesis testing, the normality of the data distribution was assessed via the Shapiro‒Wilk test, and the homogeneity of variance among groups was evaluated via Levene's test. Data that violated the assumptions of normality or homogeneity of variance were subjected to rank transformation. Specifically, raw data were converted to ranks across all observations, and the ranked data were then analyzed via conventional two-way analysis of variance (ANOVA). For data meeting parametric assumptions, two-way ANOVA was conducted via the following mixed linear model:

$$Y_{ijk}=\mu+N_i+G_j+{(N\times G)}_{ij}+\varepsilon_{ijk}$$

where *Y*_*ijk*_ is the observed dependent variable, *μ* is the overall mean, *N*_*i*_ is the fixed effect of the NE challenge, *G*_*j*_ is the fixed effect of dietary GML supplementation, (*N* × *G*)_*ij*_ is the interaction term, and *ε*_*ijk*_ is the random residual error.

When a significant interaction between two main factors was detected, Tukey's HSD test was applied for multiple pairwise comparisons. For main effects without significant interactions, simple main effects were interpreted directly from the ANOVA output. All the data are presented as the mean ± standard error of the mean (SEM), and statistical significance was set at *P* < 0.05.

## Results

### GML enhanced growth performance in broilers affected by NE

As shown in Table 3, a significant interaction was observed between dietary GML and NE infection with respect to the ADG (*P* = 0.009), which indicated that while GML had negligible effects on healthy broilers, it specifically rescued growth performance in the presence of NE challenge (*P* < 0.05). NE infection had a significant effect on ADFI and FCR, with NE broilers exhibiting decreased ADFI and increased FCR compared with noninfected broilers (*P* < 0.05).

**Table 3:** Effects of GML on growth performance in NE-challenged broilers^1^

Item	Groups^2^				SEM	*P* value		
	CON	GML	NE	GN		GML	NE	Interaction
ADFI, g/d	48.73	47.80	44.50	47.26	1.284	0.328	0.019	0.059
ADG, g/d	37.34^a^	36.62^a^	32.33^b^	35.66^a^	0.942	0.074	0.001	0.009
FCR	1.31	1.31	1.36	1.34	0.018	0.627	0.029	0.663

*ADFI* Average daily feed intake, *ADG* Average daily gain, *FCR* Feed conversion ratio

^a,^^b ^Within a row, means with no common superscripts differ significantly

^1^Means are based on 6 replicates per group with 12 birds per replicate

^2^*CON* Control, birds fed a basal diet without NE infection; *GML* Glycerol monolaurate, birds fed a basal diet containing 1,200 mg/kg GML without NE infection; *NE* Necrotic enteritis, birds fed a basal diet with NE infection; *GN* Birds fed a basal diet containing 1,200 mg/kg GML with NE infection

### GML strengthened jejunal barrier integrity in NE-challenged broilers

**Fig. 1:**
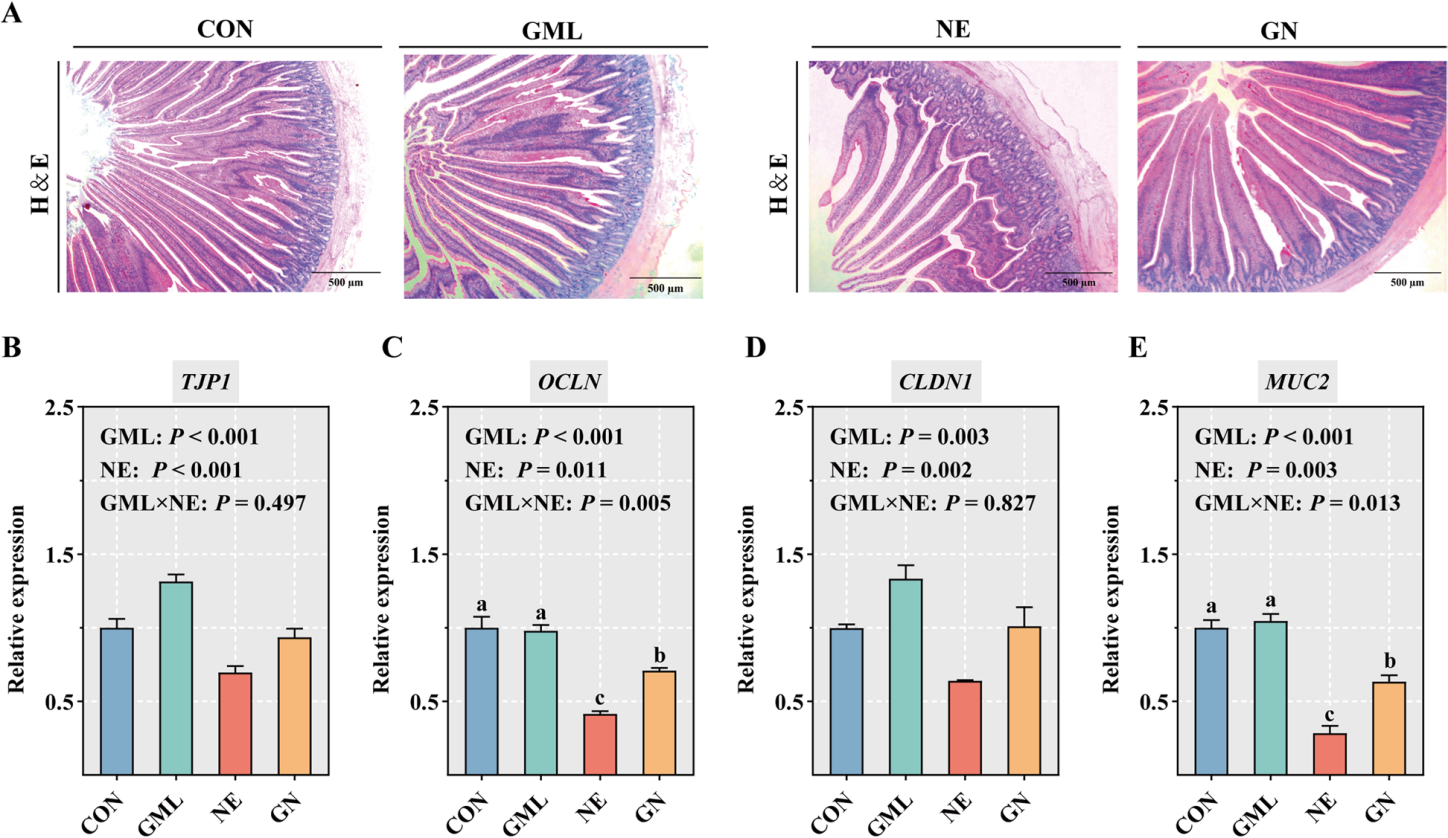
Effects of GML on the intestinal barrier function of NE broilers. **A** Representative images of HE-stained samples from the jejunum. **B**–**E** Gene expression levels of *ZO-1*, *OCLN*, *CLDN1*, and *MUC2* in the jejunum. The values are means (*n* = 6), with standard errors represented by error bars. ^a–c^Means with no common superscripts differ significantly (*P* < 0.05), two-way ANOVA with Tukey post hoc analysis. *CON* Control, birds fed a basal diet without NE infection; *GML* Glycerol monolaurate, birds fed a basal diet containing 1,200 mg/kg GML and without NE infection; *NE* Necrotic enteritis, birds fed a basal diet and with NE infection; *GN* Birds fed a basal diet containing 1,200 mg/kg GML and with NE infection; *TJP1* Zonula occludens 1, *OCLN* Occludin, *CLDN1* Claudin 1, *MUC2* Mucin 2

Table 4 and Fig. 1 present the effects of GML on intestinal tissue morphology and the expression of genes related to barrier function in NE broilers. NE infection significantly increased the intestinal lesion score and decreased the VH in the jejunum (*P* < 0.05) compared with those in the CON group. However, broilers in the GN group presented a significantly lower intestinal lesion score and an elevated jejunal VH than those in the NE group did (*P* < 0.05) (Fig. 1A). Moreover, the main effects of dietary GML on the villus/crypt ratio (*P* = 0.003) and the expression of jejunal *TJP1* (*P* < 0.001) and *CLDN1* (*P* = 0.003) were significant (Fig. 1B and D). Dietary GML improved the villus/crypt ratio and the expression of *TJP1* and *CLDN1* (*P* < 0.05). Dietary GML significantly reversed the NE-induced downregulation of the jejunal *OCLN* and *MUC2* genes (*P* < 0.05) (Fig. 1C and E).

**Table 4:** Effects of GML on intestinal morphology in NE-challenged broilers

Item	Groups^1^				SEM	*P* value		
	CON	GML	NE	GN		GML	NE	GML × NE
Intestinal lesion score	0.05^c^	0.10^c^	1.60^a^	0.80^b^	0.081	< 0.001	< 0.001	< 0.001
VH, μm	1865.00^a^	1967.00^a^	1,450.00^b^	1778.00^a^	72.290	0.047	< 0.001	0.001
CD, μm	280.60	204.00	344.10	346.30	38.450	0.208	0.002	0.184
V/C	6.65	9.57	4.01	5.50	0.813	0.003	< 0.001	0.272

*VH* Villus height, *CD* Crypt depth, *V/C* Villus height/crypt depth

^a–^^c ^Within a row, means with no common superscripts differ significantly (*n* = 6)

^1^*CON* Control, birds fed a basal diet without NE infection; *GML* Glycerol monolaurate, birds fed a basal diet containing 1,200 mg/kg GML without NE infection; *NE* Necrotic enteritis, birds fed a basal diet with NE infection; *GN* Birds fed a basal diet containing 1,200 mg/kg GML with NE infection

### GML mitigated inflammation and oxidative damage in the jejunum of NE-challenged broilers

**Fig. 2:**
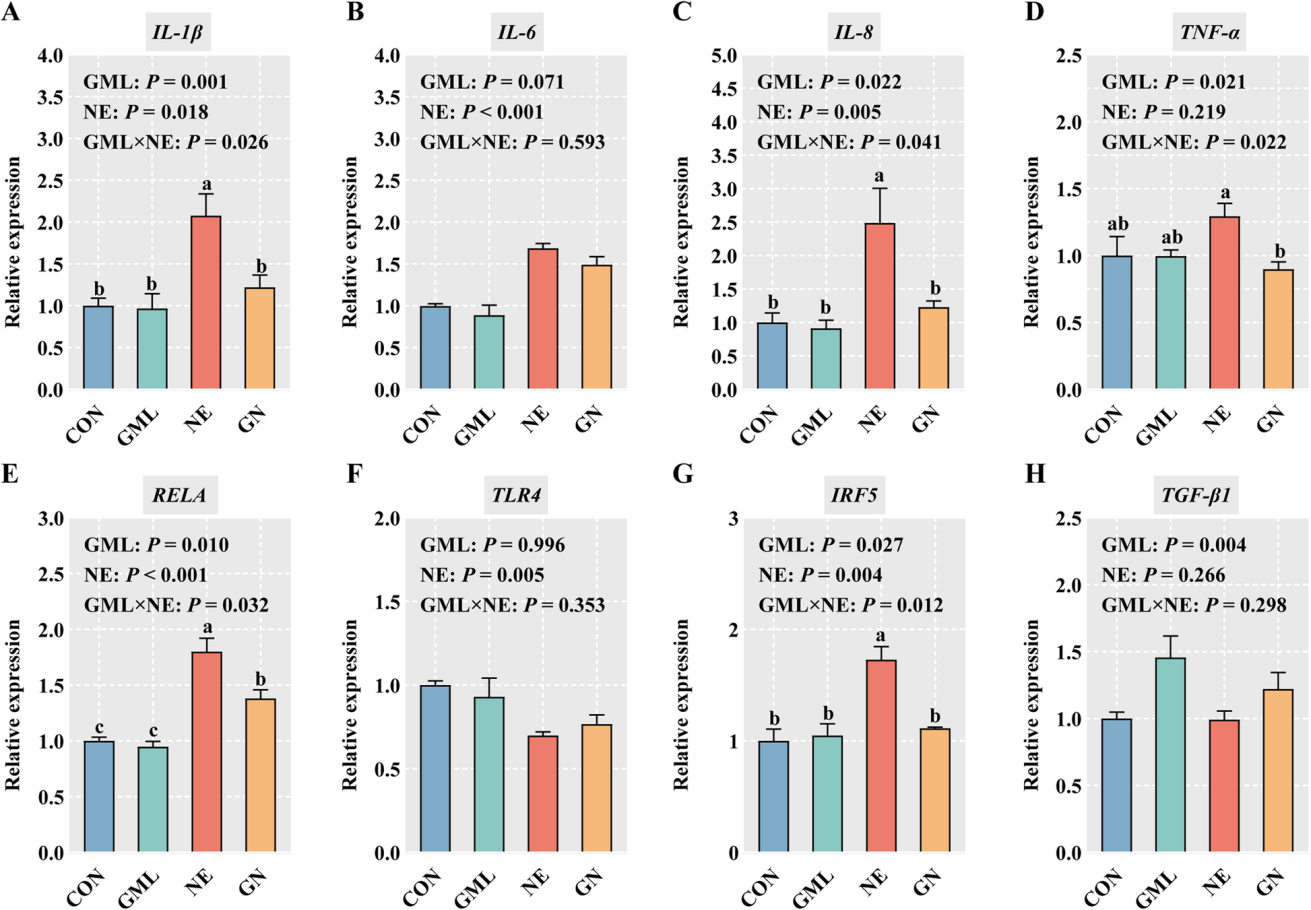
Effects of GML on jejunal inflammation in NE broilers. **A**–**H** Gene expression levels of *IL-1β*, *IL-6*, *IL-8*, *TNF-α*, *RELA*, *TLR4*, *IRF5*, and *TGF-β1* in the jejunum. The values are means (*n* = 6), with standard errors represented by error bars. ^a–c^Means with no common superscripts differ significantly (*P* < 0.05), two-way ANOVA with Tukey post hoc analysis. *CON* Control, birds fed a basal diet without NE infection; *GML* Glycerol monolaurate, birds fed a basal diet containing 1,200 mg/kg GML without NE infection; *NE* Necrotic enteritis, birds fed a basal diet with NE infection; *GN* Birds fed a basal diet containing 1,200 mg/kg GML with NE infection; *IL-1β* Interleukin 1 beta, *IL-6* Interleukin 6, *IL-8* Interleukin 8, *TNF-α* Tumor necrosis factor alpha, *RELA* Nuclear factor kappa-B p65, *TLR4* Toll-like receptor 4, *IRF5* Interferon regulatory factor 5, *TGF-β1* Transforming growth factor beta 1

The effects of the experimental treatments on the inflammatory and oxidative status are shown in Fig. 2 and Table 5. Compared with CON infection, NE infection significantly upregulated the gene expression of *IL-1β*, *IL-8*, *RELA*, and interferon regulatory factor 5 (*IRF5)* (*P* < 0.05) (Fig. 2A–G). Conversely, the expression levels of *IL-1β*, *IL-8*, *TNF-α*, *RELA*, and *IRF5* were lower in the GN group than in the NE group (*P* < 0.05). Notably, dietary GML supplementation itself exerted a significant immunomodulatory effect, as evidenced by the marked upregulation of transforming growth factor beta 1 (*TGF-β1*) expression (*P* = 0.004) (Fig. 2H). TGF-β1 is a critical cytokine for maintaining immune homeostasis and inducing mucosal tolerance. The significant increase in GML-supplemented birds, even prior to challenge, suggests that GML may proactively prime the gut environment toward an anti-inflammatory state. Moreover, dietary GML effectively alleviated the NE-induced increases in jejunal ET (*P* < 0.05) and MDA levels (*P* < 0.05), as well as the associated decrease in SOD activity (*P* < 0.05) (Table 5).

**Table 5:** Effects of GML on jejunal ET levels and oxidative status in NE-challenged broilers

Item	Groups^1^				SEM	*P* value		
	CON	GML	NE	GN		GML	NE	GML × NE
ET, EU/mg prot	4.00^b^	3.79^b^	5.34^a^	4.22^b^	0.246	< 0.001	0.047	0.031
MDA, nmol/mg prot	2.19^b^	1.92^b^	6.11^a^	2.96^b^	0.595	0.004	< 0.001	0.011
SOD, U/mg prot	145.80^b^	231.60^a^	25.30^d^	67.84^c^	7.408	< 0.001	< 0.001	0.003
CAT, U/mg prot	7.86	8.59	12.03	10.90	1.178	0.989	< 0.001	0.192

*ET* Endotoxin, *MDA* Malondialdehyde, *SOD* Superoxide dismutase, *CAT* Catalase, *prot* Protein

^a–^^d^ Within a row, means with no common superscripts differ significantly (*n* = 6)

^1^*CON* Control, birds fed a basal diet without NE infection; *GML* Glycerol monolaurate, birds fed a basal diet containing 1,200 mg/kg GML without NE infection; *NE* Necrotic enteritis, birds fed a basal diet with NE infection; *GN* Birds fed a basal diet containing 1,200 mg/kg GML with NE infection

### GML decreased ET exposure and attenuated secondary hepatic injury in NE-challenged broilers

**Fig. 3:**
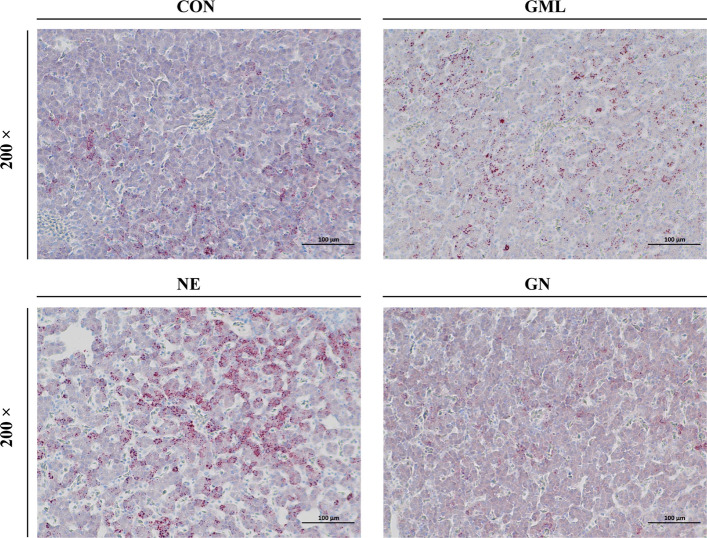
Oil Red O staining of liver sections. *CON* Control, birds fed a basal diet without NE infection; *GML* Glycerol monolaurate, birds fed a basal diet containing 1,200 mg/kg GML without NE infection; *NE* Necrotic enteritis, birds fed a basal diet with NE infection; *GN* Birds fed a basal diet containing 1,200 mg/kg GML with NE infection

Following the observation of improved intestinal barrier markers, we subsequently investigated whether this reduced the translocation of ET to the liver. As shown in Table 6, broilers in the NE group presented markedly greater serum levels of ET, IL-1β, and TNF-α than those in the CON group did (*P* < 0.05), whereas dietary GML significantly inhibited these increases in NE broilers (*P* < 0.05). Oil Red O staining revealed a disorganized liver tissue structure in the NE group of broilers, characterized by inflammatory cell infiltration and marked microvesicular steatosis (Fig. 3). This hepatic fat accumulation likely reflects secondary metabolic disturbances following intestinal inflammation and barrier dysfunction induced by NE infection. Conversely, dietary GML effectively alleviated steatosis and reduced hepatocellular damage in NE-challenged broilers, as demonstrated by a significant reduction in lipid droplets and lower serum ALT levels in the GN group than in the NE group. Significant main effects of dietary GML (*P* = 0.001) and NE infection (*P* = 0.039) on the serum AST level were observed. NE broilers presented higher serum AST levels (*P* < 0.05), whereas GML-fed broilers presented lower AST levels than the control group did (*P* < 0.05). Moreover, dietary GML significantly reversed the NE-induced increase in liver MDA content (*P* < 0.05) and increased SOD activity in NE broilers (*P* < 0.05).

**Table 6:** Effects of GML on the serum biochemical parameters and liver oxidative status of NE-challenged broilers

Item	Groups^1^				SEM	*P* value		
	CON	GML	NE	GN		GML	NE	GML × NE
Serum								
ET, EU/mL	4.77^b^	4.57^b^	5.21^a^	4.51^b^	0.118	0.036	< 0.001	0.007
IL-1β, pg/mL	24.34^b^	22.40^b^	27.75^a^	22.38^b^	0.773	< 0.001	0.006	0.004
IL-6, pg/mL	7.80	7.26	8.80	7.58	0.311	0.009	0.001	0.141
TNF-α, pg/mL	21.13^b^	20.86^b^	23.55^a^	20.31^b^	0.629	0.049	0.001	0.003
ALT, U/L	4.00^c^	4.33^c^	10.50^a^	7.08^b^	1.052	0.037	< 0.001	0.032
AST, U/L	199.50	160.40	213.80	184.80	12.880	0.001	0.039	0.583
Liver								
MDA, nmol/mg prot	5.46^b^	5.57^b^	13.79^a^	7.85^b^	1.629	0.019	< 0.001	0.015
SOD, U/mg prot	495.40^bc^	548.00^ab^	434.30^c^	588.40^a^	29.700	< 0.001	0.646	0.031
CAT, U/mg prot	11.89	11.25	11.04	10.22	0.843	0.259	0.149	0.890

*ET* Endotoxin, *IL-1β* Interleukin 1 beta, *IL-6* Interleukin 6, *TNF-α* Tumor necrosis factor alpha, A*LT* Alanine aminotransferase, *AST* Aspartate aminotransferase, *MDA* Malondialdehyde, *SOD* Superoxide dismutase, *CAT* Catalase,* prot* Protein

^a–^^c^ Within a row, means with no common superscripts differ significantly (*n* = 6)

^1^*CON* Control, birds fed a basal diet without NE infection; *GML* Glycerol monolaurate, birds fed a basal diet containing 1,200 mg/kg GML without NE infection; *NE* Necrotic enteritis, birds fed a basal diet with NE infection; *GN* Birds fed a basal diet containing 1,200 mg/kg GML with NE infection

### GML increased the diversity of the cecal microbiota in NE-challenged broilers

**Fig. 4:**
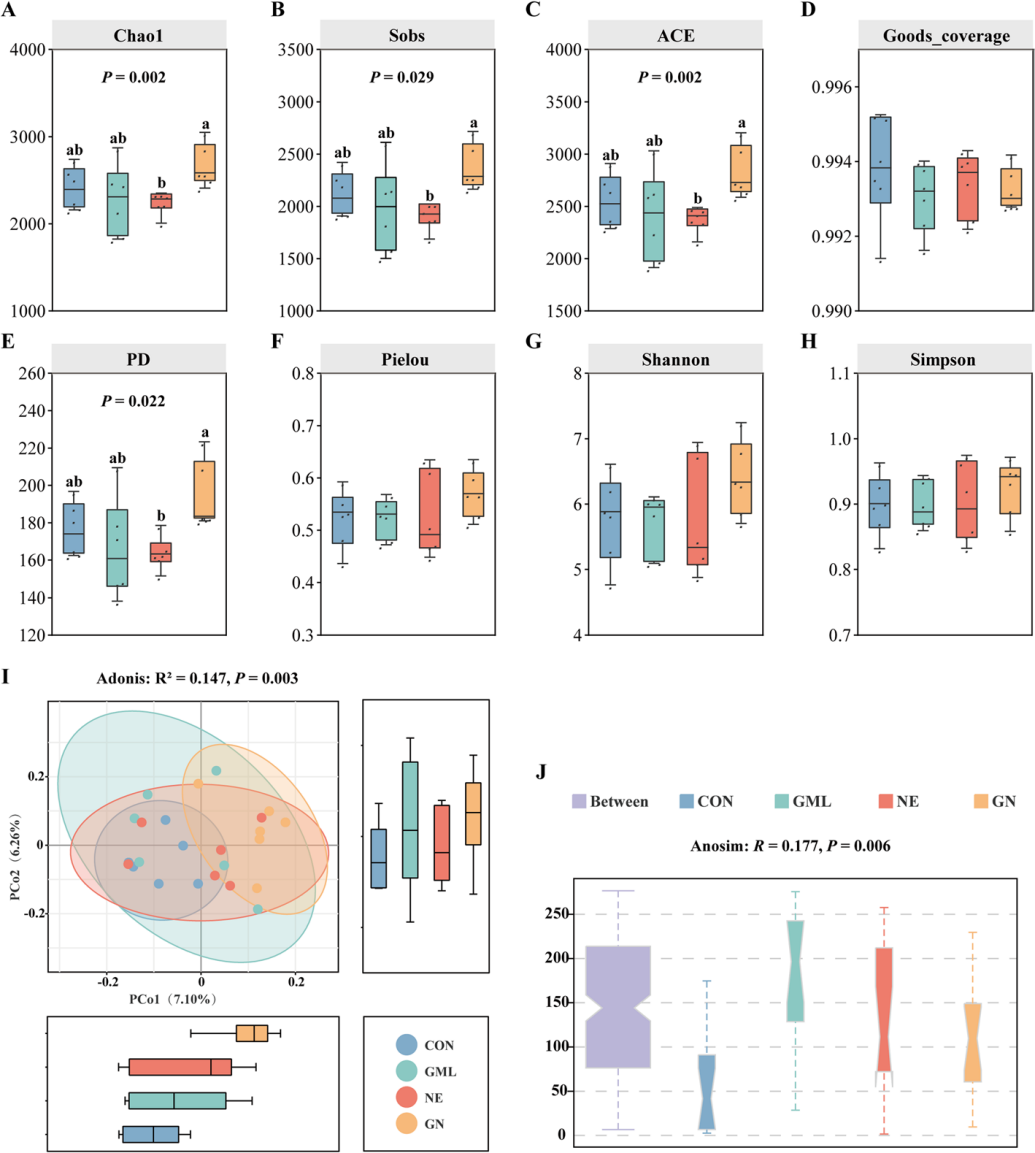
Effects of GML on microbial diversity in the cecum of NE broilers. **A**–**H** The alpha diversity indices included Chao1, Sobs, ACE, Goods_coverage, PD, Pielou, Shannon, and Simpson. **I** and **J** PCoA plot of communities based on unweighted UniFrac distances and analysis via the Adonis and ANOSIM tests. ^a,b^Means with no common superscripts differ significantly (*P* < 0.05), Kruskal‒Wallis rank sum test. The data were pooled from six birds per group (*n* = 6). *CON* Control, birds fed a basal diet without NE infection; *GML* Glycerol monolaurate; birds fed a basal diet containing 1,200 mg/kg GML without NE infection; *NE* Necrotic enteritis; birds fed a basal diet with NE infection; *GN* Birds fed a basal diet containing 1,200 mg/kg GML with NE infection

Figure 4 shows the effects of GML on alpha and beta diversity in NE broilers. The cecal microbiota of broilers in the GN group had a higher level of alpha diversity than those in the NE group, as indicated by the Chao1, Sobs, ACE, and PD indices (*P* < 0.05) (Fig. 4A–H). Beta diversity assessment based on unweighted UniFrac distances indicated that the structure of the cecal microbial community was altered by dietary GML and NE infection (Adonis: *R*^2^ = 0.147, *P* = 0.003) (Fig. 4I). ANOSIM confirmed that the distance between groups was significantly greater than the distance within groups (Anosim: *R* = 0.177, *P* = 0.006) (Fig. 4J), which indicated significant differences in the microbiome structure among the various groups.

### GML modulated the microbial community structure within the ceca of NE-challenged broilers

**Fig. 5:**
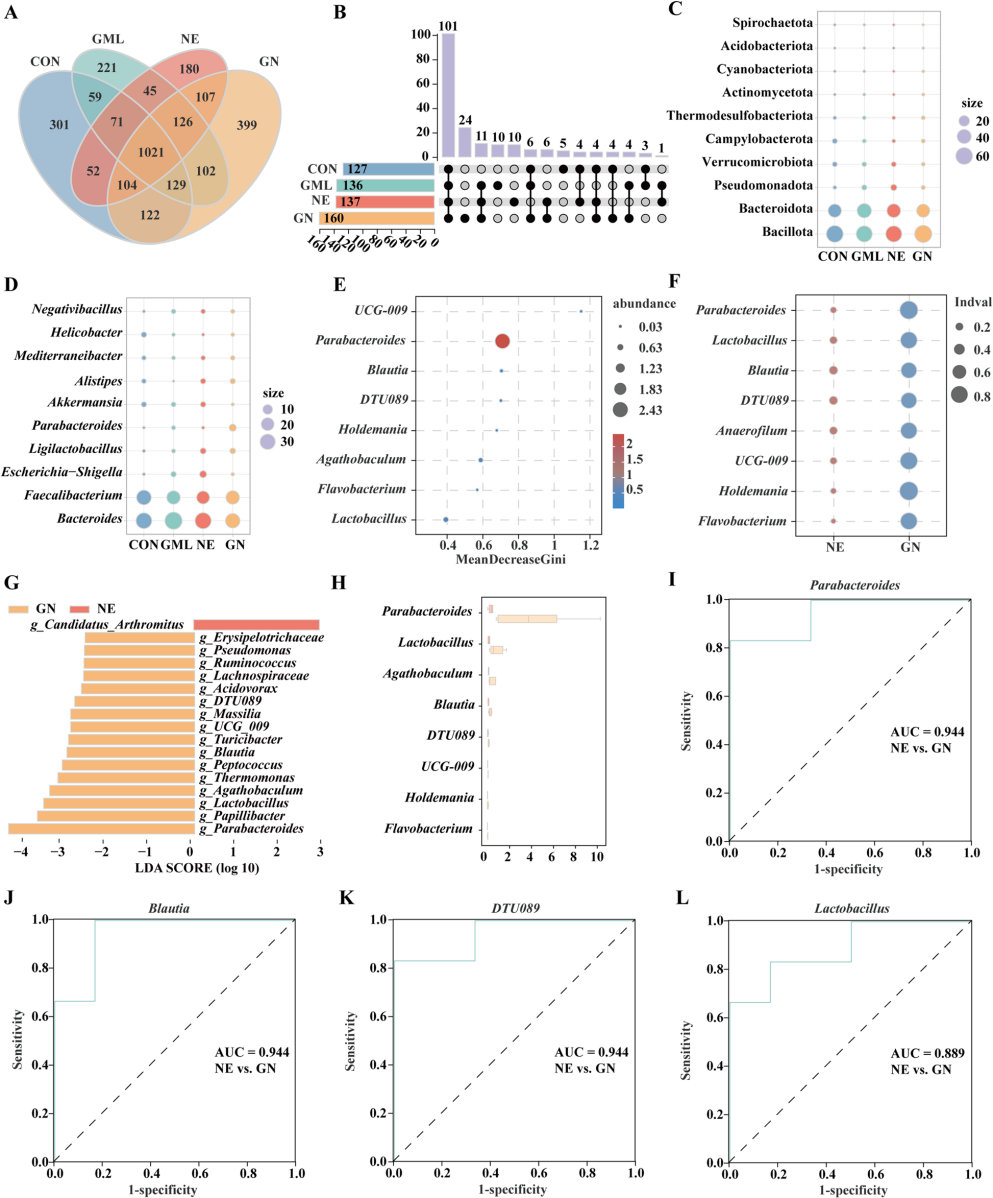
Effects of GML on the microbial community composition in the cecum of NE broilers. **A** Venn diagram at the OTU level. **B** Upset plot at the genus level. **C** and **D** Bubble chart of the relative abundance of the top 10 species at the phylum (**C**) and genus (**D**) levels. **E**–**H** Random forest analysis (**E**), indicator analysis (**F**), LEfSe analysis (**G**), and Wilcoxon rank sum test (**H**) at the genus level. **I** and **L** ROC curve analysis revealed that an AUC > 0.9 has outstanding accuracy, and 0.8–0.9 indicates excellent accuracy. The data were pooled from six birds per group (*n* = 6). *CON* Control, birds fed a basal diet without NE infection; *GML* Glycerol monolaurate, birds fed a basal diet containing 1,200 mg/kg GML without NE infection; *NE* Necrotic enteritis, birds fed a basal diet with NE infection; *GN* Birds fed a basal diet containing 1,200 mg/kg GML with NE infection

Figure 5 illustrates the microbial species composition within the ceca and presents an analysis of indicator species. The CON group presented 301 unique OTUs, whereas the GML group presented 221 unique OTUs, the NE group presented 180 unique OTUs, and the GN group presented 399 unique OTUs, with a total of 1,021 OTUs shared among these groups (Fig. 5A). At the genus level, 137 bacterial genera were exclusive to the NE group, whereas the GN group had 160 unique genera (Fig. 5B). Bubble plots revealed the mean abundance of bacterial phyla and genera across the four groups, revealing Bacillota, Bacteroidota, Pseudomonadota, and Verrucomicrobiota as the predominant phyla (Fig. 5C). Multiple bioinformatic approaches, including random forest analysis, indicator value analysis, LEfSe analysis, the Wilcoxon rank sum test, and ROC curve analysis, consistently identified *Parabacteroides*, *Lactobacillus*, *Blautia*, and *DTU089* as the key microbial biomarkers distinguishing the GN group from the NE group (AUC > 0.8) (Fig. 5D–L).

### GML alleviated inflammation and oxidative stress by altering the cecal microbiota in NE-challenged broilers

**Fig. 6:**
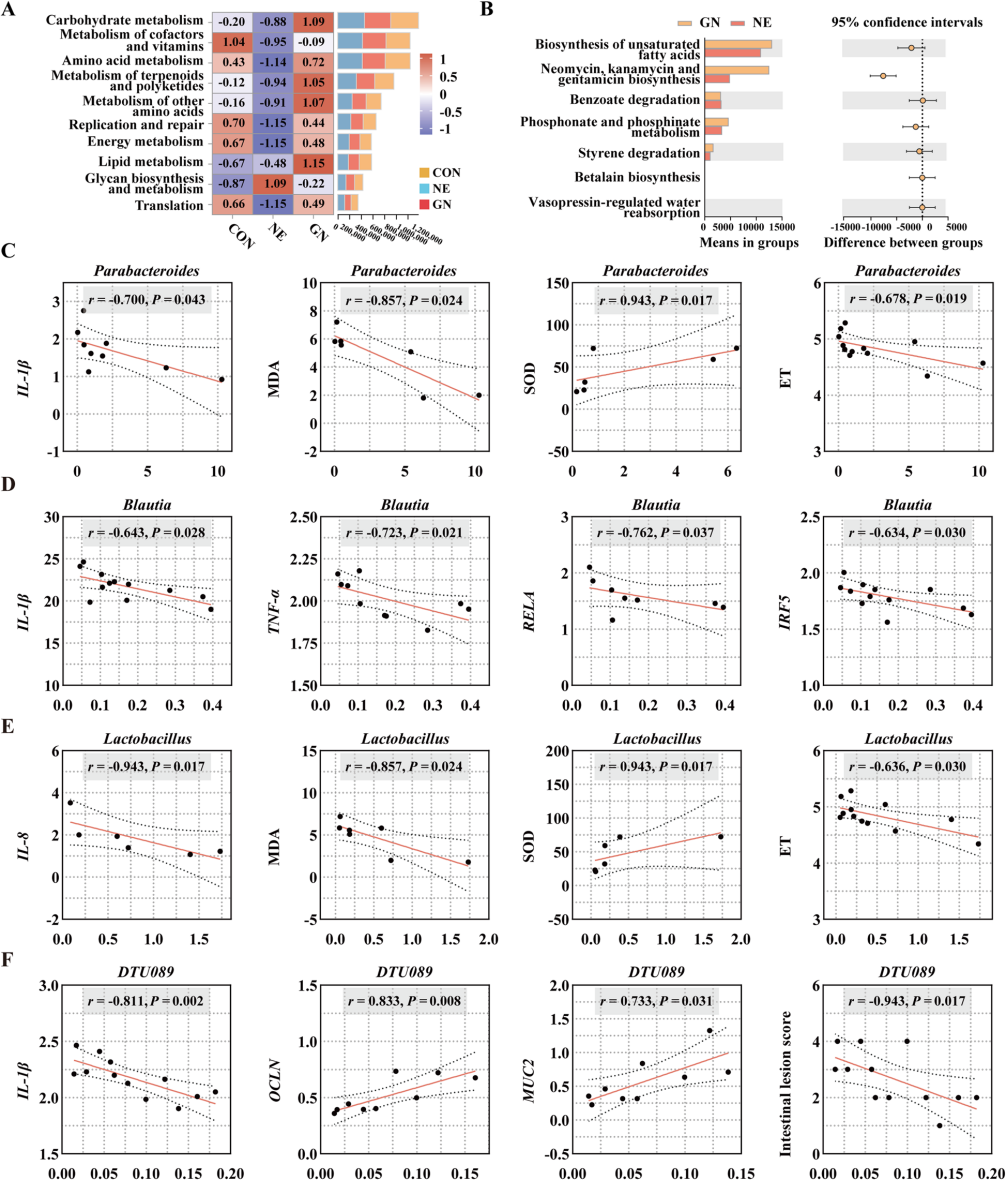
Microbial function prediction and correlation of the microbiota with inflammatory and oxidative stress parameters. **A** Top 10 enriched KEGG functions at level 2 prediction. **B** The significantly changed KEGG function at the level 3 prediction between the NE and GN groups, Welch's *t*-test. **C**–**F **Spearman correlation between differential species and parameters related to inflammation and oxidative stress. The data were pooled from six birds per group (*n* = 6). *CON* Control, birds fed a basal diet without NE infection; *GML* Glycerol monolaurate, birds fed a basal diet containing 1,200 mg/kg GML without NE infection; *NE* Necrotic enteritis, birds fed a basal diet with NE infection; *GN* Birds fed a basal diet containing 1,200 mg/kg GML with NE infection

Figure 6A depicts the categorization of KEGG pathways at level 2. Compared with those in the CON group, the functional abundances of several metabolic categories, including carbohydrate metabolism, metabolism of cofactors and vitamins, amino acid metabolism, and metabolism of terpenoids and polyketides, were lower in the NE group. However, the heatmap indicates that dietary GML effectively reversed the altered microbial functions observed in the NE broilers (Fig. 6A). Significant differences in microbial functions between the NE and GN groups were noted in the biosynthesis of unsaturated fatty acids; the biosynthesis of antibiotics such as neomycin, kanamycin, and gentamicin; benzoate degradation; phosphonate and phosphinate metabolism; and styrene degradation (Fig. 6B). *Parabacteroides* was negatively correlated with intestinal *IL-1β* gene expression (*r* = −0.700, *P* = 0.043), MDA content (*r* = −0.857, *P* = 0.024), and ET levels (*r* = −0.678, *P* = 0.019) but significantly positively correlated with SOD activity (*r* = 0.943, *P* = 0.017) (Fig. 6C). *Blautia* was significantly negatively correlated with *IL-1β* (*r* = 0.643, *P* = 0.028), *TNF-α* (*r* = −0.723, *P* = 0.021), *RELA* (*r* = −0.762, *P* = 0.037), and *IRF5* (*r* = −0.634, *P* = 0.030) expression in the jejunum (Fig. 6D). *Lactobacillus* was negatively correlated with IL-8 expression (*r* = −0.943, *P* = 0.017), the MDA content (*r* = −0.857, *P* = 0.024), and the ET level (*r* = −0.636, *P* = 0.030) but positively correlated with SOD activity (*r* = 0.943, *P* = 0.017) (Fig. 6E). Moreover, *DTU089* exhibited significant negative correlations with IL-1β (*r* = −0.811, *P* = 0.002) and jejunal lesion scores (*r* = −0.943, *P* = 0.017) but positive correlations with the expression of *OCLN* (*r* = 0.833, *P* = 0.008) and *MUC2* (*r* = 0.733, *P* = 0.031) (Fig. 6F). Spearman correlation analysis revealed that the enrichment of the beneficial genera *Parabacteroides*, *Lactobacillus*, *Blautia*, and *DTU089* was strongly associated with improved inflammatory and oxidative markers, suggesting a potential microbial role in GML-mediated protection.

## Discussion

NE is a common acute enteric infection in poultry that causes significant economic losses worldwide and raises public health concerns [8]. The most common method of inducing necrotic enteritis involves concurrent infection with coccidia and *Clostridium perfringens* [30]. In this study, NE broilers presented a reduced ADG and significant pathological changes, including intestinal congestion, hemorrhagic bruising, thinning of the intestinal wall, and intestinal swelling. Further histopathological examination of intestinal tissue confirmed the presence of villous damage in NE broilers, suggesting the successful establishment of the NE model [31]. Importantly, a limitation in the assessment of growth performance in this study should be noted. Body weight and feed intake were recorded over the entire 21 d experimental period. Therefore, the reported growth performance data represent the cumulative outcome of the basal diet, GML supplementation, coccidial vaccination, and subsequent *Clostridium perfringens* challenge. While providing an overview of production performance under these complex conditions, this design does not allow for the isolation of body weight changes attributable solely to the acute NE challenge phase. Future studies would benefit from more frequent weight measurements bracketing the specific infection period to precisely evaluate the efficacy of GML on challenge-induced growth depression. The present study highlighted the protective role of GML individually against NE, indicating improvements in growth performance and attenuation of intestinal mucosal injury in infected broilers. This efficacy can be attributed, in part, to the inherent biophysical properties of GML. As a lipophilic, amphiphilic molecule, GML readily incorporates into the lipid bilayers of Gram-positive bacteria such as *Clostridium perfringens*, disrupting membrane integrity and compromising cellular homeostasis. This direct antimicrobial effect is further supported by in vitro evidence reported by Kovanda et al., who reported that the minimum inhibitory concentration of GML against *Clostridium perfringens* strains is 300 mg/L [32]. Moreover, this antimicrobial activity may also interfere with the production or activity of key virulence factors, including alpha-toxin and NetB toxin, which are central to NE pathogenesis. Moreover, the present findings indicate that GML alleviated intestinal inflammation and oxidative stress by modulating the gut microbiota, thereby enhancing the integrity of the intestinal barrier. This improvement limited the translocation of bacterial ET and pro-inflammatory cytokines, which in turn mitigated secondary hepatic injury.

The preservation of normal villus architecture and an intact intestinal barrier are essential determinants of optimal animal growth performance [33]. Extensive research has shown that NE can compromise the integrity of the intestinal mucosa and villi, thereby negatively impacting poultry development [34]. In the present study, increased intestinal lesion scores and decreased VH were observed in NE broilers, which is in line with previous investigations [35]. Notably, dietary GML reversed the adverse effects of NE on both the intestinal lesion score and VH, highlighting the beneficial impact of GML on intestinal morphology. These improvements likely contributed to enhanced growth performance in broilers. The intestinal barrier, comprising the commensal microbiota, mucus layer, and epithelium, plays a crucial role in nutrient absorption and in protecting epithelial cells from pathogenic invasion [36]. MUC2 mucin serves as the primary structural component of the intestinal mucus barrier, which primarily functions to shield the intestinal epithelium [37]. Located underneath the mucus layer, the permeability of the intestinal epithelium is controlled by tight junction proteins [38]. These proteins are vital for the establishment and preservation of intestinal barrier integrity, with ZO-1, OCLN, and CLDN1 recognized as key contributors. Upon exposure to external stressors, the architecture of tight junctions can be compromised, leading to diminished expression of these proteins [39]. This disruption in gut homeostasis may provoke mucosal inflammation and epithelial barrier damage, thereby facilitating increased translocation of enteric bacteria and their inflammatory byproducts into the underlying tissues and systemic circulation [40]. Intestinal barrier dysfunction plays a key role in the development and progression of NE [41]. Liu et al. [16] reported significantly lower mRNA expression levels of *ZO-1*, *CLDN1*, and *MUC2* in NE broilers. Consistent with these findings, the present study demonstrated that NE infection resulted in the downregulation of *ZO-1*, *OCLN*, *CLDN1*, and *MUC2* mRNA expression. In contrast, dietary GML significantly reversed the NE-induced downregulation of jejunal *OCLN* and *MUC2* mRNA expression, suggesting that GML is beneficial for restoring mucus layer stability and maintaining tight junction integrity. The upregulation of tight junction proteins correlated with reduced intestinal permeability, as reflected by decreased ET concentrations in both the intestine and serum of the NE-challenged broilers in this study. Moreover, the assessment was conducted at a single timepoint following the NE challenge. While this captured the acute phase of intestinal injury and inflammation, it does not reveal the longer-term recovery dynamics or the potential sustained effects of GML supplementation beyond the immediate challenge period. Future studies employing multiple sequential timepoints would be valuable for delineating the temporal effects of GML on the initiation, peak, and resolution phases of NE.

A hallmark of NE is the strikingly inflammatory response that disrupts intestinal homeostasis and leads to mucosal barrier injury. Earlier studies revealed that the expression levels of various cytokines, including *IL-1β*, *IL-6*, and *TNF-α*, are elevated in NE broilers [42]. Consistent with these findings, the results of the present study demonstrated that NE infection markedly elevates the mRNA expression levels of *IL-1β*, *IL-6*, and *IL-8* in the jejunum of broilers. However, dietary GML effectively counteracted the NE-induced upregulation of *IL-1β* and *IL-8* and contributed to a decrease in *TNF-α* expression in challenged broilers. Numerous studies have underscored the capacity of GML to mitigate inflammatory responses, thereby supporting the results presented herein [43]. NF-κB is recognized as a pivotal regulator of intestinal inflammation, and its activation intensity directly correlates with inflammation severity. Previous research has revealed a significant increase in NF-κB expression in the intestines of NE broilers, which is consistent with findings from the present study [44]. Maternal GML supplementation has been shown to improve intestinal health in suckling piglets by inhibiting NF-κB signaling pathways [45]. In the present study, dietary GML substantially suppressed NF-κB p65 expression in the jejunum of NE broilers, thereby attenuating the inflammatory response. Moreover, IRF5 is a key transcription factor that drives the polarization of macrophages toward the proinflammatory M1 phenotype [46]. In the present study, dietary GML significantly reduced NE-induced *IRF5* expression in the jejunum of broilers. This suppression suggests that GML may alleviate intestinal inflammation, in part, by inhibiting the infection-triggered M1 polarization of macrophages. This interpretation is strongly supported by our previous in vitro findings, which demonstrated that GML directly promotes the polarization of chicken macrophages toward an anti-inflammatory M2 phenotype [47]. Therefore, the immunomodulatory protection conferred by GML likely involves shifting the macrophage equilibrium from a detrimental M1 state toward a protective, repair-associated M2 state.

Oxidative stress is closely associated with inflammation, both of which serve as major contributors to NE [16]. Substantial evidence indicates that the antioxidant defense system in NE-affected broilers is notably impaired [48]. In this study, the redox balance was disrupted, as indicated by increased MDA levels and decreased SOD activity. Hence, managing oxidative stress is essential for mitigating NE. Previous reports have revealed that GML enhances the antioxidant and anti-inflammatory status of weaned piglets [49]. Wang et al. [50] reported that GML increases serum antioxidant enzyme activity, thereby improving the antioxidant capacity of late-stage laying hens. Consistent with these findings, the present study demonstrated that dietary supplementation with GML reduced the jejunal MDA content and increased SOD activity in NE-infected broilers, indicating alleviation of intestinal oxidative stress.

Liver injury has become a prevalent extraintestinal manifestation associated with NE, and the hepatic inflammatory mediators triggered by such injury can further intensify intestinal inflammation [51]. Compromise of the intestinal barrier results in heightened intestinal permeability, facilitating the translocation of bacteria or harmful bacterial products from the gut lumen to the liver. Gut-derived ET is an important contributor to liver injury [52]. In this study, NE broilers presented elevated levels of ET in both the jejunum and serum, confirming a close association between gut-derived ET and inflammation in extraintestinal organs. Moreover, NE broilers presented signs of hepatic inflammatory cell infiltration and microvesicular steatosis, accompanied by significant pathological changes. This hepatic lipid accumulation is likely a direct consequence of systemic inflammation, where pro-inflammatory mediators such as TNF-α can disrupt hepatic lipid homeostasis by promoting de novo lipogenesis or impairing the assembly and secretion of very-low-density lipoproteins, thereby trapping fat within hepatocytes. Additionally, this metabolic disturbance may be exacerbated by disrupted bile acid (BA) metabolism, as *Clostridium perfringens* exhibits high bile salt hydrolase (BSH) activity that deconjugates BAs, potentially impairing lipid digestion and promoting hepatic fat deposition. The observed elevation in serum AST and ALT activities, in conjunction with these direct histopathological findings of steatosis and inflammation, indicates the presence of liver injury in the NE group [53]. In contrast, dietary GML significantly mitigated hepatic steatosis and hepatocellular injury, as evidenced by a marked reduction in lipid droplet accumulation and decreased serum ALT levels. We speculate that GML may contribute to this hepatoprotection, in part, by stabilizing the gut microbial community and the BA pool, possibly through the inhibition of BSH-producing bacteria. Consistent with these findings, Gao et al. [10] reported that GML intake alleviated heat stress-induced hepatic steatosis, diminished lipid droplet deposition, and lowered plasma AST and ALT levels. Moreover, dietary GML reduced the hepatic MDA content while increasing SOD activity in NE broilers, thereby further attenuating oxidative stress within the liver.

The maintenance of intestinal homeostasis depends on the intricate interplay among the gut microbiota, the intestinal epithelium, and the host immune system [54]. Imbalances in the gut microbiota, or dysbiosis, are strongly associated with the development of NE [55]. The findings of the present study revealed that NE infection did not significantly reduce microbial α diversity, which is consistent with previous research. However, dietary GML significantly increased the Chao 1, Sobs, ACE, and PD indices, suggesting that GML positively influences the richness of the cecal microbial community. Adonis and ANOSIM analyses revealed notable differences in community structure and β diversity across the treatment groups. To identify the principal microorganisms linked to the mitigation of NE by dietary GML, this study conducted an indicator species analysis. The intersection of the results from multiple analysis methods suggested that *Parabacteroides*, *Lactobacillus*, *Blautia*, and *DTU089* may be the key gut microorganisms responsible for the protective effects of GML against NE in broilers. Importantly, several of these genera, including *Blautia* and *Lactobacillus*, are well-established producers of short-chain fatty acids (SCFAs), such as acetate and butyrate. SCFAs serve as a primary energy source for colonocytes and are crucial for maintaining intestinal barrier function, in part by promoting the expression of tight junction proteins. Therefore, the GML-induced increases in these SCFA-producing bacteria provide a functional link to the observed increase in intestinal barrier integrity in our study. *Parabacteroides* are known to resist inflammation and promote gut health by producing acetate, which alleviates inflammation by reducing neutrophil infiltration [56]. Notably, the increase in *Parabacteroides* abundance induced by dietary GML was negatively correlated with inflammation and oxidative stress in NE broilers. *Lactobacillus* strains have been developed as probiotics and contribute to the modulation of intestinal inflammation [57]. The highly abundant and acetogenic genus *Blautia* has also been confirmed to have protective effects against intestinal inflammation [58]. In this study, an increase in the abundance of *Lactobacillus* and *Blautia* induced by GML was found to be negatively associated with inflammation-related genes and the MDA content in the jejunum of NE broilers. Dai et al. [59] demonstrated that dietary organic acids can alleviate intestinal inflammation caused by high stocking density stress in broilers by restoring the gut microbiota. Consistently, *DTU089* abundance was significantly elevated in the ceca of broilers receiving organic acid supplementation. As a monoglyceride derivative of lauric acid, GML similarly increased *DTU089* populations in NE-challenged broilers, with a strong negative correlation observed between *DTU089* abundance and *IL-1β* mRNA expression, alongside a positive correlation with genes linked to intestinal barrier integrity. Moreover, a functional prediction analysis using PICRUSt2 identified differential metabolic pathways as the predominant changes. The metabolism of nutrients was generally downregulated in NE broilers, whereas dietary GML alleviated this downregulation. Collectively, these findings indicate that GML may alleviate intestinal inflammation and oxidative damage by increasing the relative abundance of beneficial gut bacteria, thereby mitigating NE severity in broilers.

## Conclusion

In conclusion, dietary GML effectively alleviated NE in broilers by improving intestinal barrier integrity and reducing inflammation and oxidative stress, which was partially mediated through the inhibition of pro-inflammatory signaling pathways. Critically, these intestinal benefits translate into systemic protection, reducing endotoxemia and mitigating associated liver injury. The protective mechanism was associated with the restoration of microbial homeostasis, characterized by the enrichment of beneficial genera such as *Parabacteroides*, *Lactobacillus*, *Blautia*, and *DTU089*. Collectively, the present results demonstrate that GML is a viable nonantibiotic alternative that enhances gut health and resilience, offering a sustainable strategy to support intensive poultry production.

## Data Availability

No datasets were generated or analysed during the current study.

## Abbreviations

ADFI: Average daily feed intake
ADG: Average daily gain
ALT: Alanine aminotransferase
ANOSIM: Analysis of similarities
ANOVA: Analysis of variance
AST: Aspartate aminotransferase
BA: Bile acid
BSH: Bile salt hydrolase
CAT: Catalase
CD: Crypt depth
ELISA: Enzyme-linked immunosorbent assay
ET: Endotoxin
FCR: Feed conversion ratio
GAPDH: Glyceraldehyde-3-phosphate dehydrogenase
GML: Glycerol monolaurate
IL-1β: Interleukin 1β
IL-6: Interleukin 6
IRF5: Interferon regulatory factor 5
LEfSe: Linear discriminant analysis effect size
MDA: Malondialdehyde
NE: Necrotic enteritis
NF-κB: Nuclear factor kappa-B
OTUs: Operational taxonomic units
qRT-PCR: Quantitative real-time PCR
ROC: Receiver operating characteristic
SCFAs: Short-chain fatty acids
SOD: Superoxide dismutase
TNF-α: Tumor necrosis factor alpha
VH: Villus height
